# A case of erythrodermic psoriasis successfully treated with apremilast

**DOI:** 10.1111/dth.15204

**Published:** 2021-11-23

**Authors:** Matteo Megna, Sonia Sofìa Ocampo‐Garza, Gabriella Fabbrocini, Eleonora Cinelli, Angelo Ruggiero, Elisa Camela

**Affiliations:** ^1^ Section of Dermatology, Department of Clinical Medicine and Surgery University of Naples Federico II Naples Italy; ^2^ Dermatology Department Universidad Autónoma de Nuevo León, University Hospital Dr. José Eleuterio González Monterrey Mexico

Dear Editor,

Erythrodermic psoriasis (EP) is a rare condition characterized by a generalized and desquamative erythema that involves >75% of the body surface.^1^ Patients may refer systemic signs and symptoms such as malaise, fever, chills, dehydration, lymphadenopathy, arthralgia, myalgia, and tachycardia.^1^ Its prognosis is variable given the morbidity and potential mortality that may arise from electrolyte imbalance, multisystem organ failure, and sepsis.^1^ For this reason, treatment should be started promptly to increase survival. The first step includes patient stabilization through protein, fluid, and electrolytes imbalance correction and prevention of hypothermia and infections.^1^ Among systemic therapies, many drugs, either conventional, or biological, have been employed with inconstant outcomes; also, the scarcity of head‐to‐head trials on drugs comparison has limited the drawing of guidelines on EP treatment.^1^ Hence, its management should be tailored according to the patient's comorbidities, disease severity, and associated systemic signs and symptoms.^1^ Currently, biologics appear to be the most promising agents given the high safety and efficacy rates that allow their use as frontline agents, although being contraindicated in case of cancer or active infections.^1^ Conversely, conventional systemic drugs present limited efficacy and frequent flares at withdrawal. Another therapeutical opportunity is represented by apremilast, an oral, small‐molecule phosphodiesterase IV inhibitor that was approved for psoriasis in 2014.^2^ It acts by inhibiting the degradation of cyclic AMP that accumulates downregulating the expression of pro‐inflammatory cytokines.^2^ Although considered slow and less effective than biologic agents, some authors have reported successful clinical outcomes also in EP.^3^, ^4^, ^5^, ^6^, ^7^ Particularly, apremilast represents a valuable treatment for patients with specific comorbidities which contraindicate biologic use (e.g., cancer and infections).^2^ Anyway, very limited data are available on its efficacy in the management of EP, especially in long‐term. Herein we report the case of a woman affected by in situ colorectal cancer, whose EP was successfully treated with apremilast. A 64‐year‐old woman affected by psoriasis under ixekizumab came to our dermatological department for psoriasis worsening and intense pruritus. Her medical history included hypertension, and psoriasis of 20‐year‐duration for which she previously failed cyclosporine and methotrexate. Therefore, she started ixekizumab with good results at week 12. However, afterwards she experienced psoriasis worsening, intense itch, and the abrupt development of multiple Seborrheic keratosis on the back. Ixekizumab was stopped and she was prescribed blood and diagnostic tests such as thorax X‐ray and abdomen ultrasound that resulted unremarkable except for mild reduction in red blood count and fecal occult blood test positivity. Colonoscopy revealed the presence of in situ colorectal cancer that was promptly removed without significant impact on intestinal absorption. Meanwhile, psoriasis became generalized involving almost the entire body surface configuring the condition of EP (Figure 1, panels A–C). The patient was started on apremilast since it is not contraindicated in case of previous or concomitant malignancy,^2^, ^8^ and it also showed colorectal tumor regression induction in nude mice.^9^ The patient experienced a gradual subsiding of clinical manifestations reaching PASI75 at week 16 and PASI90 at 22 weeks along with itch control (Figure 1, panels D–F), maintaining this response up to 1 year of treatment. Currently, there are only six cases of EP treated with apremilast in literature (Table S1) showing variable results (three cases with complete disease control, one case with partial control, and two cases who were switched for safety issues or secondary lack of efficacy).^3^, ^4^, ^5^, ^6^, ^7^ Long‐term outcomes are reported in only case.^4^

**FIGURE 1:**
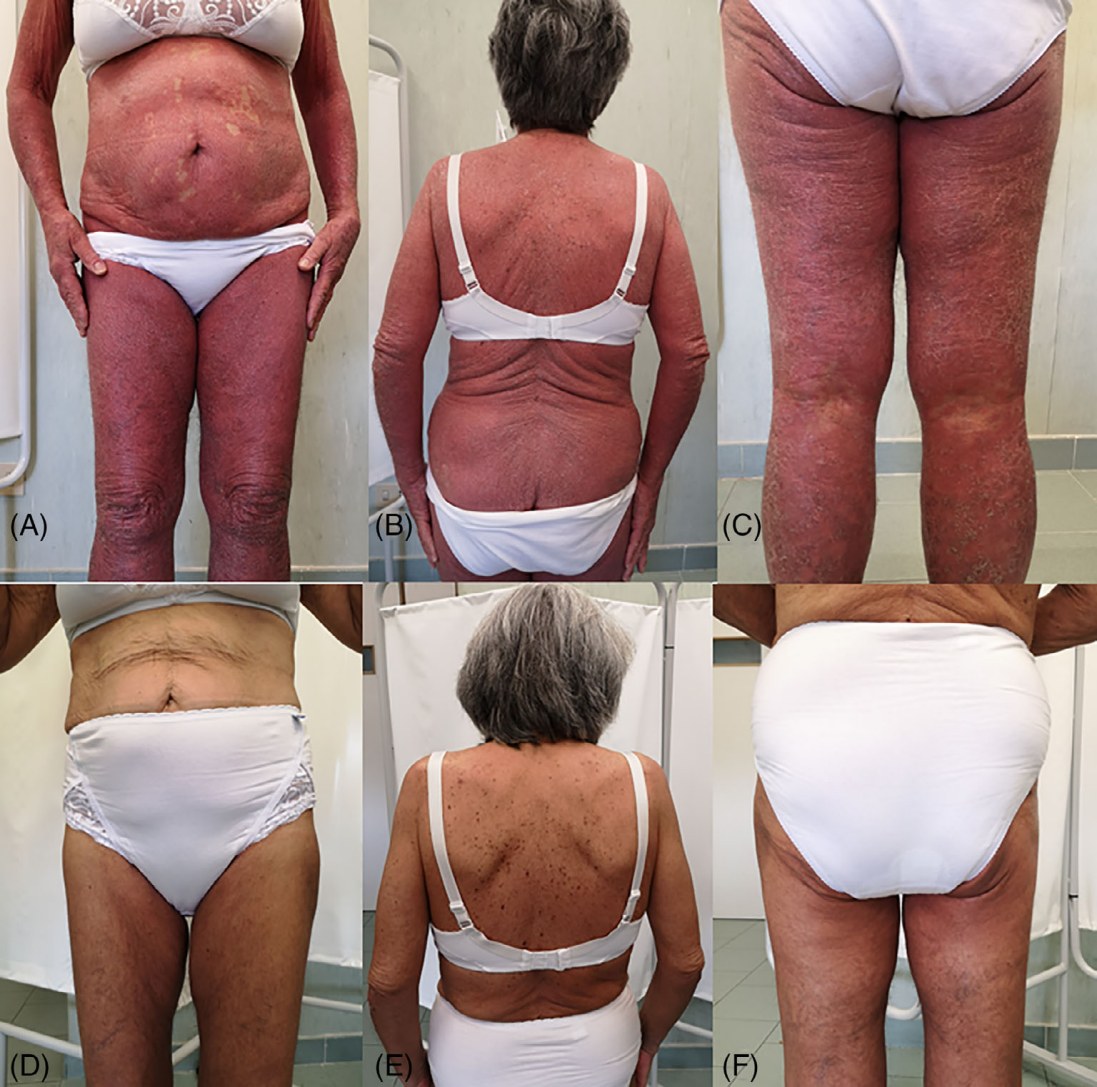
Erythrodermic psoriasis before (panels A, B, C) and after 22 weeks of apremilast treatment (panels D, E, F)

In conclusion, our case has shown that apremilast may be effective in long‐term treatment of EP, highlighting it as a precious therapeutic opportunity for patients with contraindications to other treatments. More data are needed to draw updated guidelines.

## CONFLICT OF INTEREST

The authors declare no potential conflict of interest.

## AUTHOR CONTRIBUTIONS

Matteo Megna gave substantial contribution to conception and design of the work, acquisition, analysis and interpretation of data, draft and critical revision of the article for important intellectual content, final approval of the version to be published. Sonia Sofìa Ocampo Garza gave substantial contribution to conception and design of the work, acquisition, analysis and interpretation of data, draft and critical revision of the article for important intellectual content, final approval of the version to be published. Gabriella Fabbrocini gave substantial contribution to acquisition, analysis and interpretation of data, draft and critical revision of the article for important intellectual content, final approval of the version to be published. Eleonora Cinelli gave substantial contribution to acquisition, analysis and interpretation of data, draft and critical revision of the article for important intellectual content, final approval of the version to be published. Angelo Ruggiero gave substantial contribution to conception and design of the work, draft and critical revision of the article for important intellectual content, final approval of the version to be published. Elisa Camela gave substantial contribution to conception and design of the work, acquisition, analysis and interpretation of data, draft and critical revision of the article for important intellectual content, final approval of the version to be published.

## Supporting information


**Table S1.** Review of the literature: EP cases treated with apremilast. BSA, body surface area; CsA, cyclosporin; MTX, methotrexate; PASI, psoriasis area severity index.

## Data Availability

Data sharing is not applicable to this article as no new data were created or analyzed in this study.
