# Supplementary material for: A case of erythrodermic psoriasis successfully treated with apremilast
Source: Dermatol Ther. 2021 Nov 23;35(1):e15204. doi: 10.1111/dth.15204 (PMC9286543; doi:10.1111/dth.15204)
Supplement: Supplementary file 1 — Table S1. Review of the literature: EP cases treated with apremilast. BSA, body surface area; CsA, cyclosporin; MTX, methotrexate; PASI, psoriasis area severity index. [file DTH-35-0-s001.docx]

| **Age** | **Sex** | **Comorbidities** | **Previous treatment** | **Apremilast outcomes** | **Authors** |
| --- | --- | --- | --- | --- | --- |
| 34 years | F | noncirrhotic hepatitis B, bipolar disorder, obesity, Streptococcus agalactiae and methicillin-resistant Staphylococcus aureus bacteremia, and pulmonary embolus | / | BSA decreased from 95% to 15% after 1 month | Gioe OA et al.^3^ |
| 49 years | F | iron-deficiency anemia, uterine fibroids, and severe cervical dysplasia status after total abdominal hysterectomy | CsA, MTX | BSA decreased from 65% to 3% after 1 month | Gioe OA et al.^3^ |
| 54 years | M |  | Phototherapy | PASI 44 decreased to 7.2 at week 6 and 0 at week 10 maintaining results up to 1 year treatment | Krishnamoorthy G, et al. ^4^ |
| 79 years | M | Hyprtension, sepsis | MTX, systemic steroids | PASI 44 decreased to 26.4 at day 10 then new onset atrial fibrillation and patient was switched to CsA | Arcilla J et al. ^5^ |
| 54 years | M | Hypercolesterolemia, fatty liver diasese, latent tuberculosis infection | CsA, MTX, adalimumab | PASI 49 decreased to PASI 0 after 20 days but increased to 4.8 at week 12 and 16.8 at 6 months then switched to a biologic agent | Papadavid E, at al. ^6^ |
| 45 years | M | brain oligodendroglioma, obesity, COVID 19 | All conventional and biologic drugs | PASI 45 gradually improved, allowing prednisone tapering from 50mg day to a minimal dose of 12.5 mg daily. No data on PASI improvement | Mugheddu C et al. ^7^ |
| 64 years | F | Hypertension, colorectal cancer | Ixekizuamb, CsA, MTX | PASI75 responde at week 16 and PASI90 at 22 weeks maintaining up to 1 year | Our case |

Table 1. Review of the literature: EP cases treated with apremilast. BSA= Body Surface Area; CsA= cyclosporin; MTX= methotrexate; PASI= Psoriasis Area Severity Index;
